# Cyclization of substitued 2-(2-fluorophenylazo)azines to azino[1,2-*c*]benzo[*d*][1,2,4]triazinium derivatives

**DOI:** 10.3762/bjoc.9.219

**Published:** 2013-09-16

**Authors:** Aleksandra Jankowiak, Emilia Obijalska, Piotr Kaszynski

**Affiliations:** 1 Organic Materials Research Group, Department of Chemistry, Vanderbilt University, Nashville, TN 37235, USA; 2 Faculty of Chemistry, University of Łódź, Tamka 12, 91403 Łódź, Poland

**Keywords:** DFT, heterocycles, mechanism, synthesis

## Abstract

Light-induced cyclization of several substituted 2-(2-fluorophenylazo)azines in the presence of Ca^2+^ ions to the corresponding triazinium derivatives is investigated experimentally and computationally. The azo derivatives of 4-methylpyridine **4** undergo facile cyclization to the corresponding triazinium **1**, and the rate of cyclization increases with increasing number of fluorine atoms at the benzene ring. No triazinium ions were obtained from azo derivatives of 4-cyanopyridine, pyrazine and pyrimidine, presumably due to their instability under the reaction conditions. The experimental results and mechanism are discussed with the aid of DFT computational results.

## Introduction

**Figure 1:**
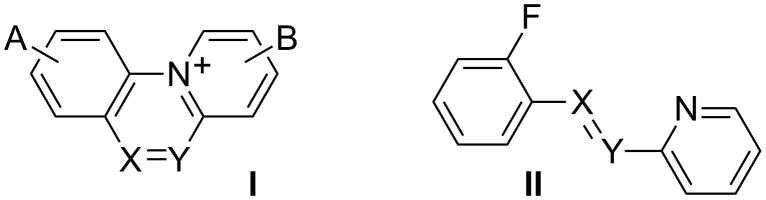
Structures of cations **I** and precursors **II**.

Derivatives of benzo[*c*]quinolizinium [1–4] (**I**, X = Y = CH, Figure 1) and its di-aza analogue pyrido[2,1-*c*]benzo[*d*][1,2,4]triazinium (**I**, X = Y = N) belong to a class of tricyclic cations featuring general structure **I** and are of general interest because of their biological activity. For instance, it was demonstrated that the parent cation **1a** intercalates into the minor grove of DNA [5] and to be cytotoxic to cisplatinum-resistant tumor cell lines [6]. Several of its derivatives containing Cl, Br, Me and OMe substituents at the benzene ring were prepared by acid-catalyzed photoinduced cyclization of phenylazopyridines [5]. Recently, we demonstrated an efficient alternative method for the formation of **1a** and other parent tricyclic azinium cations of general structure **I** (A = B = H) by light-induced intramolecular cyclization of fluorides **II** (Figure 1) in the presence of Ca^2+^ ions [7].

**Figure 2:**
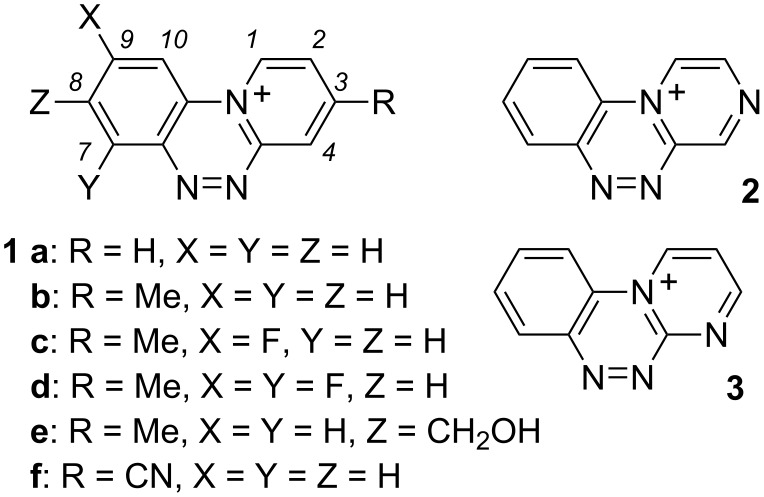
Structures of triazinium cations **1**–**3**.

A further progress in the investigation of cation **1a** and its applications as a pharmacophore or a component of organic materials requires access to functionalized derivatives, in which substituents control the properties and allow the incorporation into more complex molecular structures. As the first step towards these goals we tested the suitability of our previously described method [7] for the synthesis of several derivatives of **1a** and also for two other azines. Initially we selected CH_3_, CN and F substituents to investigate the reactivity of the azines, and CH_2_OH to provide a potential synthetic handle for the incorporation into more complex structures. Here we present the synthesis of four substituted derivatives of **1a** and investigate the preparation of cations derived from pyrazine **2** and pyrimidine **3** shown in Figure 2. Our experimental results are augmented with DFT computational results.

## Results and Discussion

### Cyclization and cation formation

**Scheme 1:**
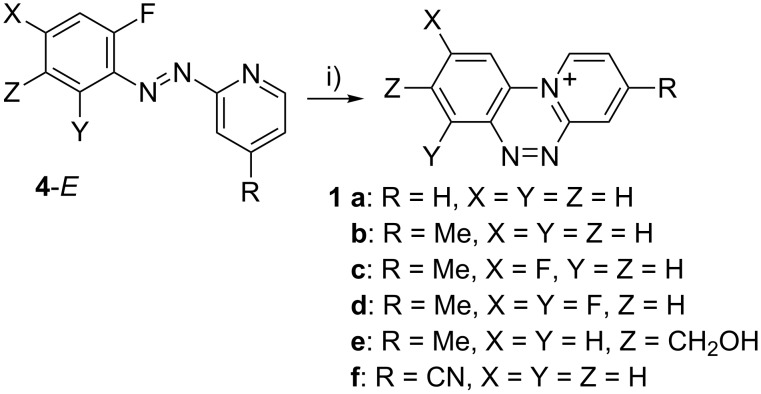
Synthesis of triazinium cations **1**. Reagents and conditions: i) *h*ν (halogen lamp or sunlight), Ca^2+^, MeCN/H_2_O (9:1).

**Figure 3:**
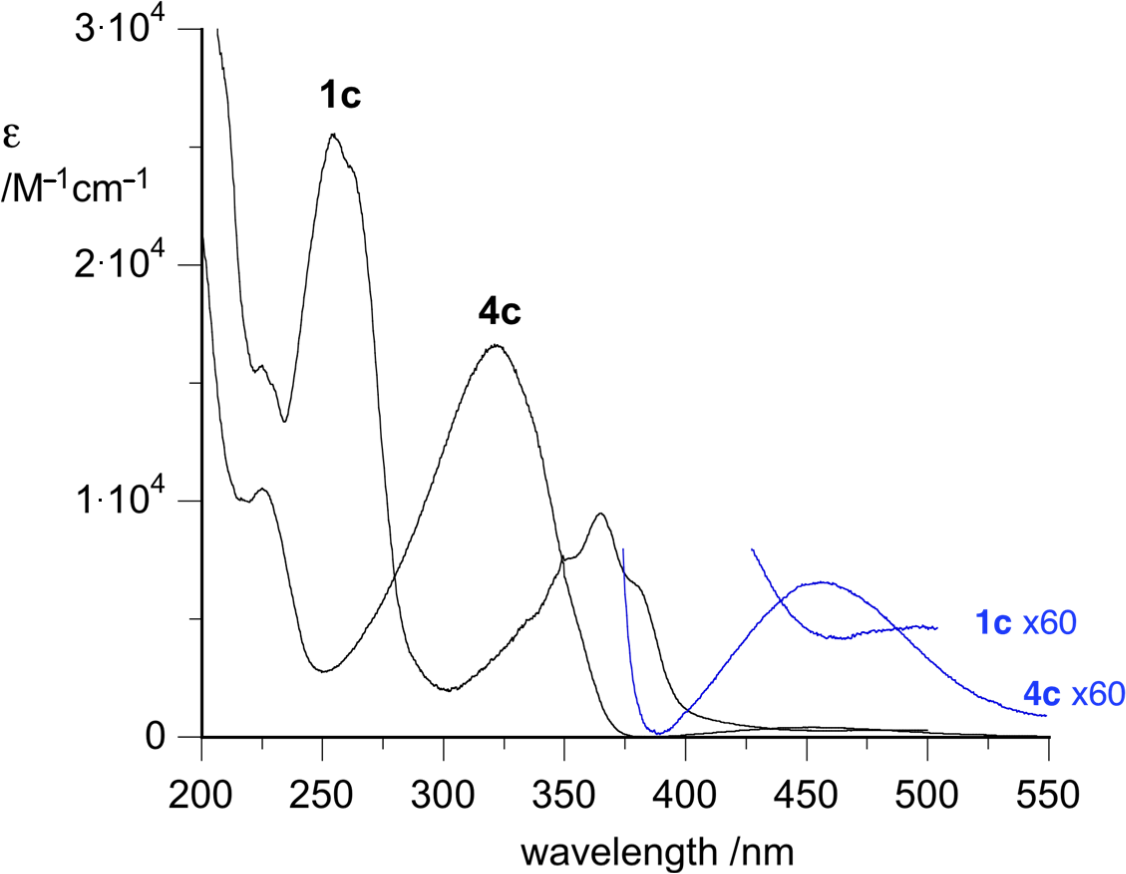
Electronic absorption spectra for **1c** and **4c** (MeCN). Blue lines represent magnified areas of the spectra.

Salts **1** were prepared by photocyclization of the corresponding azo derivatives **4** in the presence of Ca^2+^ ions in aqueous MeCN (Scheme 1). Diazenes **4** show weak absorption bands in the visible (e.g. 452 nm, log ε = 2.6 for **4c**), while salts **1** exhibit only a weak tailing absorption above 400 nm, as shown for **4c** and **1c** in Figure 3. Therefore irradiation was effectively conducted in Pyrex vessels.

The cyclization of 2-fluorophenylazopyridines **4b** and **4e** was conducted using a 500 W halogen lamp in the presence of Ca(OTs)_2_ [7], and cations **1b** and **1e** were isolated as tosylates in 75% and 95% yield, respectively. Under these conditions diazenes **4c** and **4d** underwent cyclization, but the resulting cations **1c** and **1d** were unstable and partially decomposed during the reaction. Therefore, their cyclization was carried out with sunlight and tosylate **1c** was isolated in pure form in 90% yield. In contrast, cation **1d** partially decomposed during the workup, presumably due to the activation by means of the fluorine atoms and their electron-withdrawing effect. A pure sample of **1d** could not be isolated. A comparison of the cyclization rates of the three fluorophenylazo derivatives **4b**–**4d** was conducted in NMR tubes exposed to sunlight. The experiment demonstrated that the rate of cyclization increases with increasing number of fluorine atoms, and after 1 h conversion of **4b** to **1b** was 30%, **4c** to cation **1c** 80%, and **4d** to **1d** was complete. Full conversion of **4b** to **1b** was achieved after 5 h. It should be pointed out that the cyclization and formation of the cations requires both light and Ca^2+^ ions, and no product formation was observed if one of them is absent. While in most experiments Ca(OTs)_2_ served as a source of Ca^2+^, in some other CaCl_2_ was used, and the Cl^−^ anion was later replaced by TsO^−^.

**Figure 4:**
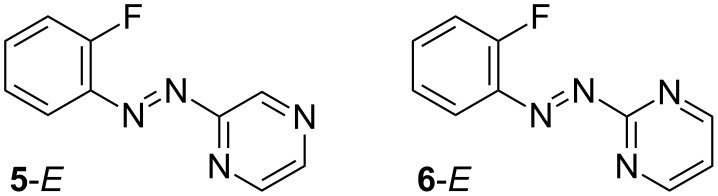
Strcutures of *trans* azo derivatives **5***-E* and **6***-E*.

Attempts to cyclize the cyano derivative **4f**, using a halogen lamp were unsuccessful and after 3 h only decomposition products were observed by ^1^H NMR along with some residual starting **4f**. No characteristic NMR pattern that could be ascribed to the cation **1f** was detected. Similarly unsuccessful were the attempts to cyclize azopyrazine **5** (halogen lamp/ice bath or sunlight, Figure 4) and azopyrimidine **6** (halogen lamp/reflux); instead complex mixtures of products were obtained.

### Synthesis of azo precursors

**Scheme 2:**
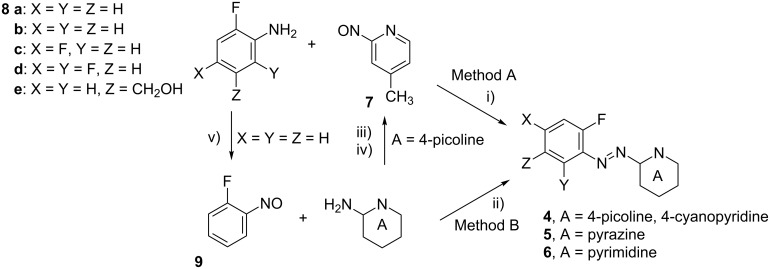
Synthesis of azo precursors. Reagents and conditions: i) AcOH cat, CH_2_Cl_2_, rt, 25 h; ii) toluene, 50% NaOH, 50 °C, 25 min; iii) NCS, Me_2_S, CH_2_Cl_2_, –20 °C; iv) mCPBA, CH_2_Cl_2_, 0 °C; v) Oxone^®^, CH_2_Cl_2_/H_2_O rt, 25 h.

Azo compounds **4b**–**4e** were prepared in 38%–90% yield using method A (Scheme 2) in which 2-nitroso-4-picoline (**7**) [8] was condensed with 2-fluoroaniline (**8a**) or its derivatives **8c**–**8e**. The 2-fluoro-5-hydroxymethylaniline (**8e**) was obtained according to a literature procedure [9]. 4-Cyano derivative **4f** was prepared in 50% yield by method B [7,10] involving condensation of 2-amino-4-cyanopyridine with 1-fluoro-2-nitrosobenzene (**9**) [7]. Azoazines **5** and **6** were obtained in a similar way by condensation of **9** with 2-aminopyrazine and 2-aminopyrimidine, respectively, and their preparation was described before [11].

Interestingly, the trifluoro derivative **4d** had exceptionally limited thermal stability: After heating at 60 °C in CDCl_3_ it was converted to another species also containing the picoline fragment, according to the ^1^H NMR analysis. The new compound did not undergo photocyclization and was not analyzed further.

### Mechanistic and computational analyses

**Scheme 3:**
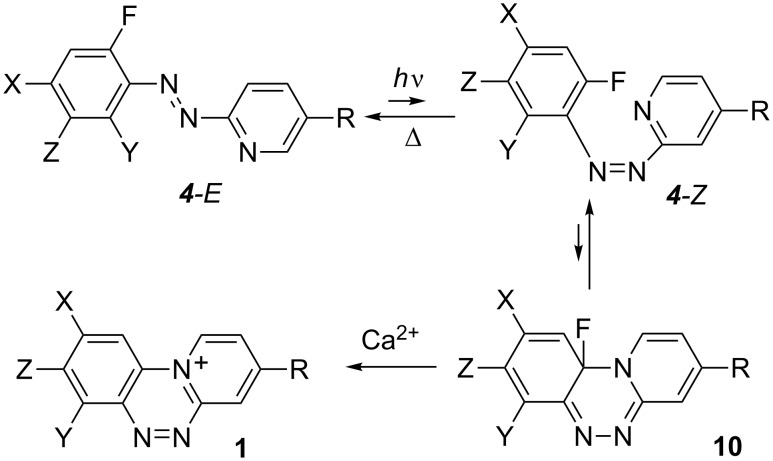
Formation of cations **1** from diazenes **4**.

The proposed mechanism for the formation of the triazinium cations from fluorophenylazoazines involves a photo-induced *trans*-to-*cis* isomerization of the azo group followed by Ca^2+^-assisted cyclization (Scheme 3) [7], which, in principle, can proceed either thermally or photochemically. Thus, the success of the reaction depends on i) the efficient access to the *Z*-isomer, and ii) the relative rates of cyclization vs back isomerization to the *E*-isomer. The supply of the requisite *Z*-isomers of the azoazines is best accomplished by photoisomerization of the *E*-isomers, since the *Z*-isomers cannot be populated thermally due to the high *E* → *Z* thermal isomerization barrier (Δ*G*^‡^_298_ > 35 kcal/mol) [7]. The *Z*-forms however, thermally and also photochemically isomerize back to the more stable *E*-isomers, and the rates are substituent depended [12]. Thus, with the broadband irradiation a photostationary state establishes providing a constant ratio of *Z*- to *E*-isomers.

Previous analyses suggested [7] that the cyclization of **4***-Z* involves 6 π electrons leading to non-aromatic product **10**, which upon Ca^2+^-assisted departure of the fluoride undergoes aromatization and formation of cation **1**. In this work, the theoretical analysis is concentrated on the effect of substituents and on the type of azine on the formation of the cations. Therefore the cyclization of *cis*-azoazines **4-***Z*–**6-***Z* was investigated at B3LYP/6-311+G(2d,p)//B3LYP/6-311G(2d,p) level of theory in MeCN as dielectric medium without participation of metal ions. The computational studies were expanded by inclusion of **4g-***Z*– **4i-***Z* for a better understanding of the structure–reactivity relationship. The resulting free energy differences between the *Z*-isomers, relevant to the cyclization process, and the cyclic products, and free energy of activation for a series of diazenes are listed in Table 1 and Table 2.

**Table 1:** Calculated free energy activation and change (kcal/mol) for the cyclization of *cis*-azopyridine **4***-Z* to the nonaromatic product **10** in MeCN (Scheme 3).^a^

	R	X	Y	Z	Δ*G*^‡^_298_^b^	Δ*G*_298_^c^

**a**	H	H	H	H	22.4	5.7
**b**	Me	H	H	H	23.1	5.7
**c**	Me	F	H	H	18.5	2.4
**d**	Me	F	F	H	18.9	4.6
**f**	CN	H	H	H	23.3	5.2
**g**	OMe	H	H	H	23.5	4.5
**h**	Me	H	H	OMe	23.2	3.9
**i**	Me	H	H	CN	19.0	4.8

^a^Calculations at B3LYP/6-311+G(2d,p)//B3LYP/6-311G(2d,p) level with MeCN as dielectric medium. ^b^Energy of the transition state **10-TS**. ^c^Energy difference between product **10** and azopyridine **4-***Z*.

**Table 2:** Calculated free energy activation and change (kcal/mol) for cyclization of *cis*-azoazines to the nonaromatic product in MeCN.^a^

reaction	Δ*G*^‡^_298_^b^	Δ*G*_298_^c^

**4a-***Z* → **10a**	22.4	5.7
**5-***Z* → **11**	18.7	2.7
**6-***Z* → **12**	25.0	7.5

^a^Calculations at B3LYP/6-311+G(2d,p)//B3LYP/6-311G(2d,p) level in MeCN as dielectric medium. ^b^Energy of the transition state. ^c^Energy difference between the cyclic product and azoazine.

The results in Table 1 and Table 2 show that the free energy of activation, Δ*G*^‡^_298_, required for the cyclization of all considered azoazines is in a range of 18–25 kcal/mol, and the process is endergonic by about 5 kcal/mol. Thus, for all investigated compounds the cyclization is thermally accessible and also thermally reversible at ambient temperature. These computational results are consistent with experimental observations, that all azoazines reacted, however only 5 cations (**1a**–**1e**) were isolated. The remaining cations, **1f**, **2** and **3**, could have been formed, but were presumably unstable under the reaction conditions, as already observed for **1d**. Also consistent with the computational results is the observation that the formation of **1** requires a fluoride scavenger, apparently for the aromatization of the cyclic product.

**Figure 5:**
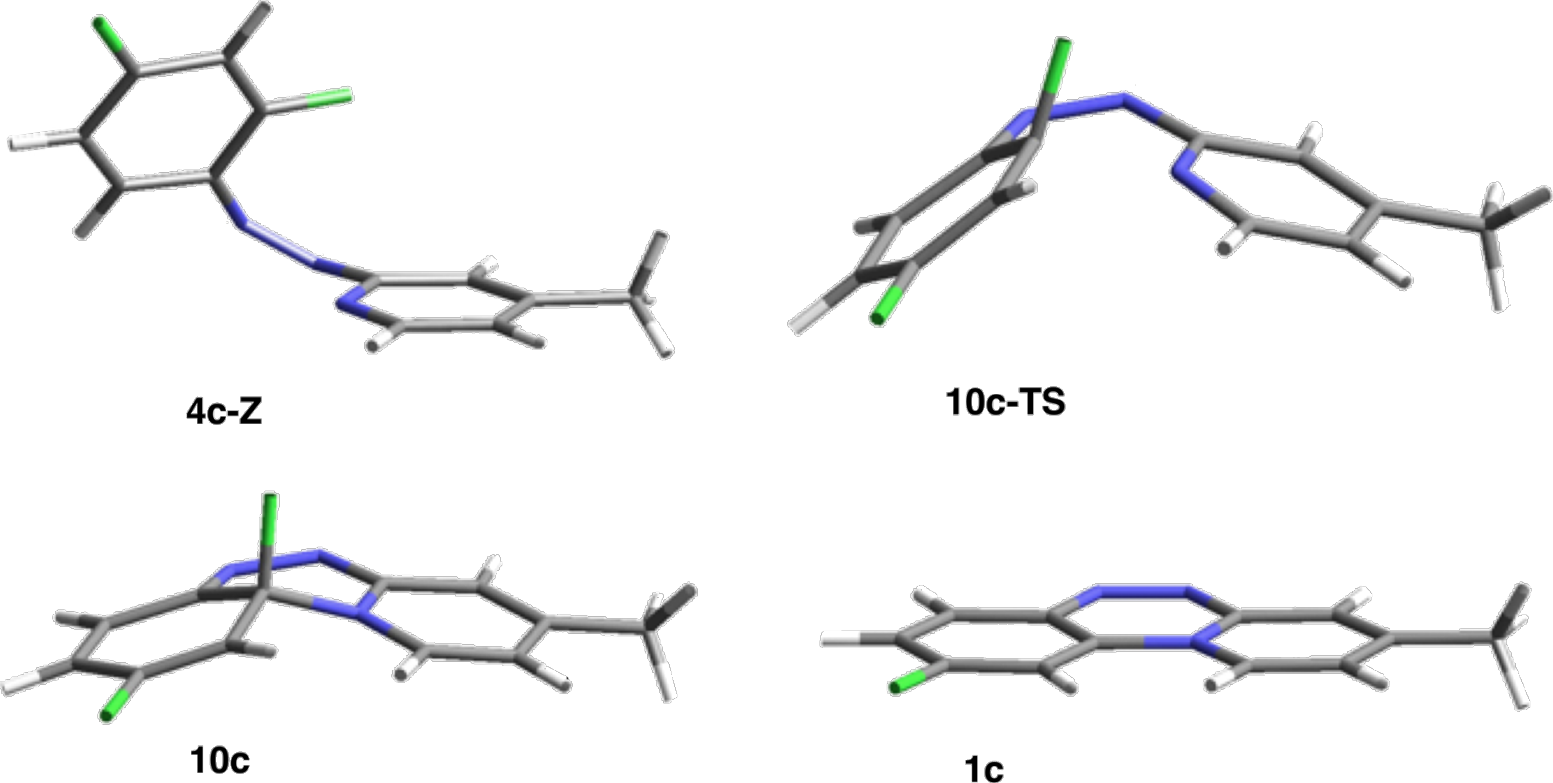
B3LYP/6-311G(2d,p)-optimized geometries for structures involved in cyclization of **4c** to **1c**.

The analysis of species involved in the cyclization demonstrates that the C^…^N and C–F distances change similarly for all azoazines in the process regardless of the substituent. Thus, the C^…^N distance decreases by 1.232 Å from 3.150 ± 0.03 Å in the *cis-*azoazine to 1.918 ± 0.009 Å in the transition state (TS) and then to 1.457 ± 0.004 Å in the cycloadduct, as shown for the cyclization of **4c** to **10c** in Figure 5. At the same time the C–F distance increases only by about 2% from 1.350 ± 0.002 Å in the *cis*-azoazine to 1.374 ± 0.003 Å in the TS. A more significant increase in the C–F distance, by 7%, is observed in the cyclic product (1.445 ± 0.005 Å).

A detailed analysis of the DFT results for the azopyridine series demonstrates that the rate of cyclization only correlates with the character of the substituent at the benzene ring and does not depend on the pyridine ring. For a simple nucleophilic aromatic *ipso*-substitution reaction (NAS) electron-donating groups in the C(4) position of the pyridine ring in **4** should increase the reaction rate. Computational results demonstrate the contrary and as the electron donation of the substituent R increases in the order H (**4a**) < CH_3_ (**4b**) < OCH_3_ (**4g**) so does the activation energy (Table 1). In contrast, increasing electron withdrawing ability of the substituent Z in the *para*-position to the leaving fluorine atom increases the reaction rate as observed in a series **4h** (Z = OCH_3_, Δ*G*^‡^_298_ = 23.2 kcal/mol), **4a** (Z = H, Δ*G*^‡^_298_ = 22.4 kcal/mol), and **4i** (Z = CN, Δ*G*^‡^_298_ = 19.0 kcal/mol) in Table 1.

**Figure 6:**
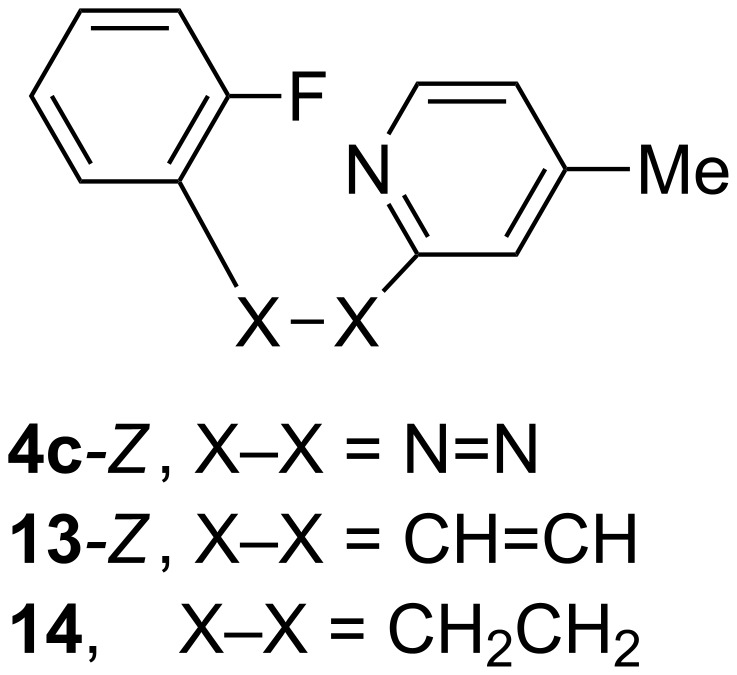
Structures of three close analogues.

For further probing of the cyclization mechanism, the mildly activating [13–14] N=N-bridging group in **4c**–*Z* was replaced by the non-activating CH=CH group in **13-***Z* (Figure 6). A computational analysis demonstrated that this structural modification resulted in a modest increase of activation energy Δ*G*^‡^_298_ for the cyclization by 3.1 kcal/mol. However, replacement of the bridging π-bond with the aliphatic CH_2_CH_2_ group in compound **14** prevents the cyclization process, and the analogous cyclic product could not be located on the potential energy surface. These results indicate that the cyclization process involves 6 π electrons, which allows the formation of the non-zwitterionic product such as **10**. Comparison of cyclization of **4c-***Z* and **13-***Z* further demonstrates that the activation energy is lower for the azene than for the analogues stilbene, which presumably is related to the electronegativity of the bridging group.

The analysis of the data of three fluoro derivatives **4b**–**4d** in Table 1 demonstrates that the addition of one or two fluorine atoms at the benzene ring in **4b** significantly lowers the activation barrier for the cyclization, by about 4.5 kcal/mol, for **4c-*Z*** and **4d-*Z***, which is consistent with the accellerated formation of cations **1c** and **1d** observed experimentally. Interestingly, a bigger effect is predicted for the 2,4-difluorophenyl derivative **4c** than for the 2,4,6-trifluorophenyl analogue **4d**.

Among the three parent azoazines the least unfavorable cyclization is calculated for the pyrazine derivative **5-***Z* (Δ*G*_298_ = 2.7 kcal/mol), which also has the lowest TS energy (Δ*G*^‡^_298_ = 18.7 kcal/mol, Table 2). On the other hand, the cyclization of pyrimidine **6-***Z* is most endergonic and has the highest TS energy among all azoazines considered in this work. These significant differences between the three parent azoazines can be explained with lower aromaticity of the pyrazine ring [15], and with the electropositive character of the N(1) position of the pyrimidine ring, when compared to the pyridine analogue. These two factors may also contribute to the higher reactivity of cations **2** and **3** and hence their apparent instability under the reaction conditions.

## Conclusion

A DFT computational analysis indicates that the cyclization of *cis*-fluorophenylazoazines can occur thermally at room temperature for all considered compounds. The reaction is endergonic with 2.4–7.5 kcal/mol, and the formation of the triazinium cations is accomplished by aromatization of the cyclic product by Ca^2+^ ion-assisted removal of the fluoride. These results are supported by experimental data, which demonstrate a facile conversion of all azoazines under the reaction conditions, although only 5 out of 8 triazinium salts were isolated. The remaining three, all of them containing a CN substituent and derived from pyrazine and pyrimidine, are presumably too reactive under the reaction conditions to be isolated in pure form.

A brief computational investigation of the mechanism demonstrates the importance of the unsaturated bridge in the cyclization step and points towards a 6π-electrocyclization rather than to a simple nucleophilic aromatic displacement mechanism.

Among the triazinium salts prepared in this work is the hydroxymethyl derivative **1e**, which, in principle, can be incorporated into more complex molecular structures.

## Computational Details

The quantum-mechanical calculations were carried out at B3LYP/6-311G(2d,p) level of theory using the Gaussian 09 suite of programs [16]. The geometry optimizations were undertaken using tight convergence limits and with no symmetry constraints. Vibrational frequencies were used to characterize the nature of the stationary points. Zero-point energy (ZPE) corrections were scaled by 0.9806 [17]. Transition state structures for cyclization reactions were located using the STQN method [18] requested with the QST2 keyword and default convergence criteria. Final energies for each optimized structure were calculated with the B3LYP/6-311+G(2d,p)//B3LYP/6-311G(2d,p) method in MeCN as dielectric medium using the IPCM model [19] requested with the SCRF=IPCM keyword (epsilon = 36.64).

## Experimental

### General remarks

Melting points are uncorrected. NMR spectra were recorded at 300 MHz (^1^H) and 100 MHz (^13^C) in CD_3_CN, DMSO-*d*_6_ or CDCl_3_ and chemical shifts are refered to the solvent peaks (1.96, 2.54 and 7.26 ppm, respectively).

### Photocyclization and preparation of salts **1**. General Porcedure

**Method A** [7]: Azo compound **4** (1.0 mmol) and calcium *p*-toluenesulfonate (190 mg 0.5 mmol) or calcium chloride (56 mg, 0.5 mmol) were dissolved in a mixture of MeCN/H_2_O (9:1, 30 mL). The resulted solution was irradiated with a 500 W halogen lamp and gently refluxed until TLC control showed full conversion of the substrate (about 1.5 h). The solvents were evaporated. The residue was dried in a desiccator over P_2_O_5_ (12 h). The solid was washed with CH_2_Cl_2_, the residue was dissolved in hot MeCN and filtered. The solvent was evaporated and the crude product was recrystallized from aqueous MeCN.

**Method B**: The reaction mixture prepared as in method A was exposed to intense sunlight, while stirring until TLC control showed full conversion of the substrate (about 1.5 h). The work-up of the reaction mixture was as described in method A.

**3-Methylpyrido[2,1-*c*][1,2,4]benzotriazin-11-ium *p*-toluenesulfonate (1b)**: Method A, yield 95%: ^1^H NMR (300 MHz, CD_3_CN) δ 2.33 (s, 3H), 2.98 (s, 3H), 7.16 (d, *J* = 7.8 Hz, 2H), 7.60 (d, *J* = 8.0 Hz, 2H), 8.35 (t, *J* = 7.8 Hz, 1H), 8.43 (d, *J* = 7.5 Hz, 1H), 8.49 (t, *J* = 8.1 Hz, 1H), 8.83 (d, *J* = 8.8 Hz, 1H), 9.03 (d, *J* = 8.0 Hz, 1H), 9.12 (s, 1H), 9.87 (d, *J* = 7.0 Hz, 1H); HRMS *m*/*z* calcd for C_12_H_10_N_3_, 196.0869; found, 196.0892.

**9-Fluoro-3-methylpyrido[2,1-*c*][1,2,4]benzotriazin-11-ium *p*-toluenesulfonate (1c)**: Method B, yield 90%: mp >190 °C dec.; ^1^H NMR (300 MHz, CD_3_CN) δ 2.35 (s, 3H), 2.96 (s, 3H), 7.19 (d, *J* = 7.5 Hz, 2H), 7.60 (d, *J* = 7.1 Hz, 2H), 8.11 (t, *J* = 7.8 Hz, 1H), 8.44 (d, *J* = 6.7 Hz, 1H), 8.64 (dd, *J*_1_ = 9.2 Hz, *J*_2_ = 2.2 Hz, 1H), 9.00–9.60 (m, 1H), 9.11 (s, 1H), 9.73 (d, *J* = 6.8 Hz, 1H); ^13^C NMR (100 MHz, DMSO-*d*_6_) δ 20.8, 21.8, 104.1 (d, *J* = 30 Hz), 121.8 (d, *J* = 25 Hz), 125.5, 128.0, 129.7, 130.2, 132.3, 135.7 (d, *J* = 11 Hz), 137.5, 138.9, 141.9, 145.8, 160.6; UV–vis (MeCN) λ_max_ (log ε) 225 (4.20), 254 (4.41), 262 sh (4.38), 365 (3.98), 379 sh (3.81); anal. calcd for C_19_H_16_FN_3_O_3_S: C, 59.21; H, 4.18; N, 10.90; found: C, 58.92; H, 4.09; N, 10.95.

**7,9-Difluoro-3-methylpyrido[2,1-*c*][1,2,4]benzotriazin-11-ium chloride (1d):** Method B, yield 40–80% (based on NMR): ^1^H NMR (300 MHz, CD_3_CN) δ 2.98 (s, 3H), 7.99 (t, *J* = 8.1 Hz, 1H), 8.49 (d, *J* = 5.5 Hz, 1H), 8.56 (d, *J* = 8.9 Hz, 1H), 9.16 (s, 1H), 9.77 (d, *J* = 6.8 Hz, 1H).

**8-Hydroxymethyl-3-methylpyrido[2,1-*c*][1,2,4]benzotriazin-11-ium *p*-toluenesulfonate (1e)**: Method A, yield 75%: mp >150 °C dec.; ^1^H NMR (300 MHz, DMSO-*d*_6_) δ 2.32 (s, 3H), 2.98 (s, 3H), 4.97 (d, *J* = 5.0 Hz, 2H), 5.92 (t, *J* = 5.5 Hz, 1H), 7.14 (d, *J* = 7.8 Hz, 2H), 7.50 (d, *J* = 8.0 Hz, 2H), 8.49 (d, *J* = 8.5 Hz, 1H), 8.64 (d, *J* = 5.5 Hz, 1H), 8.95 (s, 1H), 9.17 (d, *J* = 8.9 Hz, 1H), 9.35 (s, 1H), 10.36 (d, *J* = 6.8 Hz, 1H); ^13^C NMR (100 MHz, DMSO-*d*_6_) δ 20.8, 21.6, 61.6, 116.4, 121.8, 125.5, 128.0, 128.1, 129.6, 130.2, 131.9, 136.6, 137.6, 141.2, 142.0, 145.7, 148.2, 159.2; anal. calcd for C_20_H_19_N_3_O_4_S: C, 60.44; H, 4.82; N, 10.57; found: C, 60.15; H, 4.78; N, 10.47.

### Azo compounds **4**. A general procedure

Method A. To the solution of amine (1.0 mmol) in dry CH_2_Cl_2_ (2 mL), 2-nitroso-4-picoline (**7**, 1.0 mmol) was added followed by a catalytic amount (1 drop) of acetic acid. The reaction mixture was stirred at rt for 24 h, protected from a light. The solvent was evaporated and the residue was purified on a silica gel plug (CH_2_Cl_2_/EtOAc, 5:1) to give the corresponding azo compound as orange-red crystals. Analytically pure samples were obtained by recrystallization (hexane/CH_2_Cl_2_).

**2-(2-Fluorophenylazo)picoline (4b)**: Yield: 83%: mp 78–80 °C; ^1^H NMR (300 MHz, CDCl_3_) δ 2.48 (s, 3H), 7.18–7.34 (m, 3H), 7.48–7.56 (m, 1H), 7.64 (s, 1H), 7.90 (t, *J* = 7.7 Hz, 1H), 8.61 (d, *J* = 4.4 Hz, 1H); ^13^C NMR (100 MHz, CDCl_3_) δ 21.2, 114.5, 117.1 (d, *J* = 20 Hz), 117.9, 124.3 (d, *J* = 4 Hz), 126.5, 133.7 (d, *J* = 4 Hz), 140.4 (d, *J* = 6 Hz), 149.3, 149.8, 160.7 (d, *J* = 258 Hz), 163.3; anal. calcd for C_12_H_10_FN_3_: C, 66.97; H, 4.68; N, 19.52; found: C, 66.93; H, 4.66; N, 19.41.

**2-(2,4-Difluorophenylazo)picoline (4c):** Yield 90%: mp 99–101 °C; ^1^H NMR (300 MHz, CD_3_CN) δ 2.48 (s, 3H), 7.14 (t, *J* = 8.8 Hz, 1H), 7.26 (ddd, *J*_1_ = 11.3 Hz, *J*_2_ = 8.7 Hz, *J*_3_ = 2.6 Hz, 1H), 7.37 (d, *J* = 5.0 Hz, 1H), 7.60 (s, 1H), 7.91 (dd, *J*_1_ = 15.3 Hz, *J*_2_ = 8.8 Hz, 1H), 8.58 (d, *J* = 4.8 Hz, 1H); ^1^H NMR (300 MHz, CDCl_3_) δ 2.48 (s, 3H), 6.92–7.08 (m, 2H), 7.26 (d, 1H), 7.63 (s, 1H), 7.94 (d, *J* = 8.0 Hz, 1H), 7.99 (d, *J* = 8.6 Hz, 1H), 8.60 (d, *J* = 4.6 Hz, 1H); ^13^C NMR (100 MHz, CDCl_3_) δ 21.1, 105.1 (t, *J* = 24 Hz), 112.0 (dd, *J*_1_ = 27 Hz, *J*_2_ = 4 Hz), 114.5, 119.3 (d, *J* = 10 Hz), 126.5, 137.3 (dd, *J*_1_ = 7 Hz, *J*_2_ = 4 Hz), 149.2, 149.8, 161.2 (dd, *J*_1_ = 260 Hz, *J*_2_ = 7 Hz),163.1, 165.3 (dd, *J*_1_ = 255 Hz, *J*_2_ = 12 Hz); UV–vis (MeCN) λ_max_ (log ε) 225 (4.02), 322 (4.22), 541 (6.61); anal. calcd for C_12_H_9_F_2_N_3_: C, 61.80; H, 3.89; N, 18.02; found: C, 61.79; H, 3.79; N, 17.92.

**2-(2,4,6-Trifluorophenylazo)picoline (4d):** Yield 38%: ^1^H NMR (300 MHz, CD_3_CN) δ 2.49 (s, 3H), 6.85 (t, *J* = 8.8 Hz, 2H), 7.26 (d, 1H), 7.56 (s, 1H), 8.58 (d, *J* = 4.9 Hz, 1H); ^1^H NMR (300 MHz, CDCl_3_) δ 2.48 (s, 3H), 7.09 (t, *J* = 9.3 Hz, 2H), 7.26 (d, 1H), 7.61 (s, 1H), 8.61 (d, *J* = 4.8 Hz, 1H); HRMS *m*/*z* calcd for C_12_H_9_F_3_N_2_, 252.0749; found, 252.0738.

**2-(2-Fluoro-5-hydroxymethylphenylazo)picoline (4e)**: Yield 69%: mp 154–155 °C; ^1^H NMR (300 MHz, CDCl_3_) δ 2.48 (s, 3H), 4.73 (s, 2H), 7.28–7.32 (m, 1H), 7.52–7.60 (m, 1H), 7.64 (s, 1H), 7.88 (d, *J* = 7.1 Hz, 1H), 7.60 (d, *J* = 4.9 Hz, 1H); ^13^C NMR (100 MHz, CDCl_3_) δ 21.2, 64.0, 114.3, 115.9, 117.2 (d, *J* = 20 Hz), 126.6, 132.2 (d, *J* = 8 Hz), 137.6 (d, *J* = 4 Hz), 140.0 (d, *J* = 7 Hz), 149.1, 150.0, 160.0 (d, *J* = 257 Hz), 163.1; anal. calcd for C_13_H_12_FN_3_O: C, 63.66; H, 4.93; N, 17.13; found: C, 63.94; H, 4.88; N, 17.20.

**4-Cyano-2-(2-fluorophenylazo)pyridine (4f):** Method B. 4-Cyano-2-aminopyridine (238 mg, 2.0 mmol) was dissolved in toluene (3 mL) and a 50% aqueous solution of NaOH (1.5 mL) and 2-fluoronitrosobenzene [7] (**9**, 293 mg, 2.3 mmol) was added. The mixture was vigorously stirred at 50 °C for 25 min. After cooling, water was added, and the mixture was extracted with CH_2_Cl_2_. Extracts were dried over Na_2_SO_4_, and the solvent was evaporated. The residue was purified by column chromatography (SiO_2_, hexane/CH_2_Cl_2_) to give 230 mg (50% yield) of azo derivative **4f** as an orange solid: ^1^H NMR (300 MHz, CD_3_CN) δ 7.34 (t, *J* = 7.8 Hz, 1H), 7.41 (t, *J* = 9.7 Hz, 1H), 7.62– 7.72 (m, 1H), 7.76–7.88 (m, 2H), 8.02 (s, 1H), 8.89 (d, *J* = 4.9 Hz, 1H); ^1^H NMR (300 MHz, CDCl_3_) δ 7.27–7.41 (m, 2H), 7.56–7.61 (m, 1H), 7.66 (d, *J* = 5.0 Hz, 1H), 8.05 (s, 1H), 8.93 (d, *J* = 4.9 Hz, 1H); ^13^C NMR (100 MHz, CD_3_CN) δ, 116.8, 117.0, 118.2, 118.3, 123.2, 125.6 (d, *J* = 6 Hz), 127.9, 135.9 (d, *J* = 8 Hz), 140.7 (d, *J* = 7 Hz), 151.4, 161.3 (d, *J* = 257 Hz), 164.0; anal. calcd for C_12_H_7_FN_4_: C, 63.71; H, 3.12; N, 24.77; found: C, 63.81; H, 3.14; N, 24.61.

## Supporting Information

File 1 General methods and synthetic procedures.
