# Supplementary material for: Cyclization of substitued 2-(2-fluorophenylazo)azines to azino[1,2-c]benzo[d][1,2,4]triazinium derivatives
Source: Beilstein J Org Chem. 2013 Sep 16;9:1873–80. doi: 10.3762/bjoc.9.219 (PMC3778383; doi:10.3762/bjoc.9.219)
Supplement: File 1 — General methods and synthetic procedures. [file Beilstein_J_Org_Chem-09-1873-s001.pdf]

Cyclization of substitued 2-(2-fluorophenylazo)azines to azino[1,2-*c*]benzo[*d*][1,2,4]triazinium derivatives

Aleksandra Jankowiak[1],[a] Emilia Obijalska[1],[a §] and Piotr Kaszynski [*1,2]

Address: [1]Organic Materials Research Group, Department of Chemistry, Vanderbilt University, Nashville, TN 37235 USA and [2]Faculty of Chemistry, University of Łódź, Tamka 12, 91403 Łódź, Poland

Email: Piotr Kaszynski - piotr.kaszynski@vanderbilt.edu

* Corresponding author

[§]A visiting student from the laboratory of Professor Grzegorz Mlostoń, Univeristy of Łódź, Poland.

General methods and synthetic procedures

Table of Content:

1. Additional synthetic procedures ….S2
2. ^{1}H NMR spectra for selected compounds ….S3
3. Selected interatomic distances ….S5
4. Archive data for selected DFT calculations ….S5
5. References ….S8

1. Additional synthetic procedures

3-Amino-4-fluorobenzoic acid [1]. Following a literature procedure [2], 4-fluoro-3-nitrobenzoic acid (1.0 g, 5.4 mmol) was reduced with hydrogen in the presence of Pd/C (60 mg, 10% on charcoal) in EtOH (10 ml) over 24 h. The reaction mixture was passed through Celite, the filtrate was evaporated, and the residue was purified on a silica gel plug (EtOAc) to give 0.75 g (90% yield) of 3-amino-4-fluorobenzoic acid as a white solid: ^{1}H NMR (300 MHz, CD_3CN) δ 4.40 (bs, 1H), 7.05 (dd, J_1 = 10.8 Hz, J_2 = 8.6 Hz, 1H), 7.26–7.35 (m, 1H), 7.46 (dd, J_1 = 8.8 Hz, J_2 = 1.7 Hz, 1H).

3-Amino-4-fluorobenzyl alcohol (9e) [3]. Following literature procedure [3] a solution of 3-amino-4-fluorobenzoic acid (4.0 g, 26.3 mmol) in dry THF (20 ml) was treated with 1 M solution of BH_3 in THF (53 mmol, 53 mL) at 0 °C. The reaction mixture was stirred at rt for 24 h, MeOH (10 mL) was slowly added, and stirred for 15 min, the solvents were evaporated, and the residue was purified on a silica gel plug (CH_2Cl_2/ EtOAc, 1:1) to give 3.47 g (94% yield) of alcohol **9e** as a white solid: mp 68–70 °C; ^{1}H NMR (300 MHz, $CDCl_3$) δ 3.74 (bs, 2H), 4.56 (s, 2H), 6.62–6.70 (m, 1H), 6.79 (d, J = 8.6 Hz, 1H), 6.95 (dd, J_1 = 10.7 Hz, J_2 = 8.4 Hz, 1H).

2. ^{1}H NMR Spectra for selected compounds.

a) recrystallized tosylate of **1b** in CD_3CN.

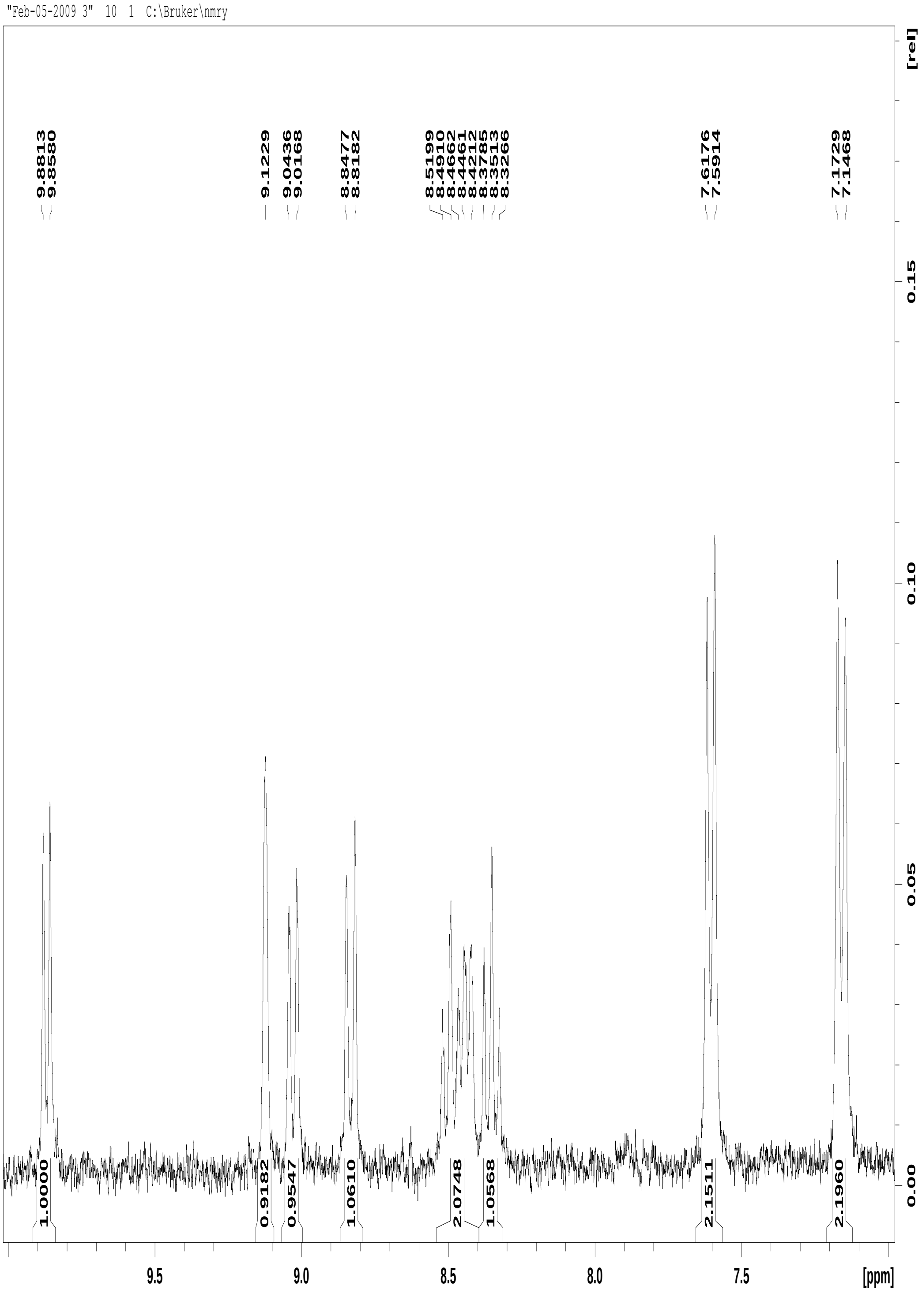

b) Crude reaction mixture of **4d** in the presence of $CaCl_2$ in CD_3CN.

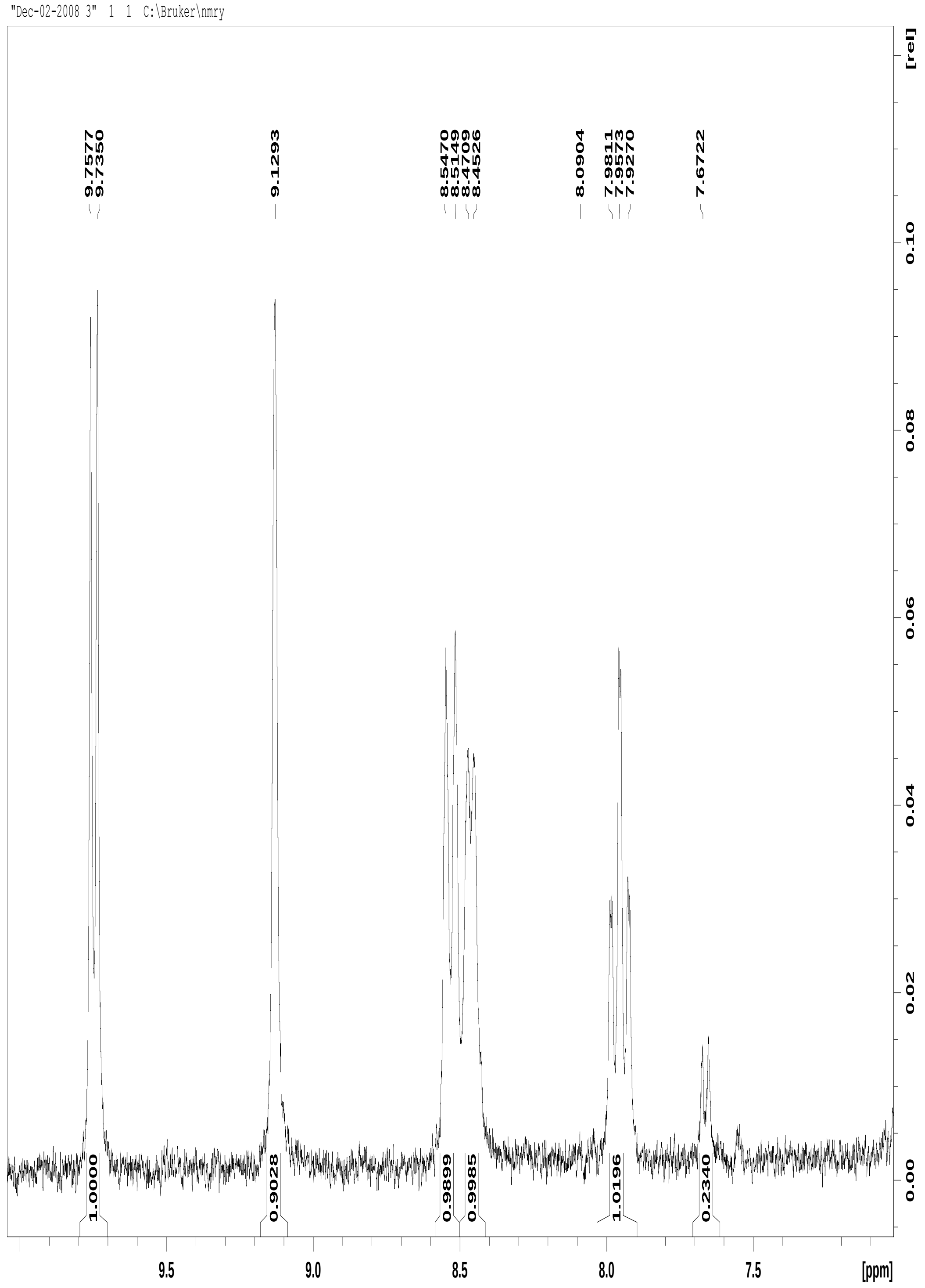

3. Selected interatomic distances

Table S1: Selected interatomic distanes (Å) for species involved in the formation of triazinium from azoazines.[a]

	Azopyridines				*cis*-azoazine		TS		cycloadduct	
	R	X	Y	Z	C–F	C···N	C–F	C···N	C–F	C–N
**a**	H	H	H	H	1.351	3.151	1.375	1.916	1.446	1.458
**b**	Me	H	H	H	1.351	3.149	1.375	1.918	1.448	1.457
**c**	Me	F	H	H	1.347	3.101	1.373	1.928	1.447	1.459
**d**	Me	F	F	H	1.346	3.160	1.371	1.930	1.445	1.461
**f**	CN	H	H	H	1.351	3.166	1.371	1.917	1.439	1.456
**g**	OMe	H	H	H	1.351	3.146	1.376	1.922	1.449	1.457
**h**	Me	H	H	OMe	1.353	3.158	1.379	1.900	1.452	1.449
**i**	Me	H	H	CN	1.345	3.116	1.370	1.927	1.441	1.462
azopyrazine					1.351	3.161	1.372	1.910	1.441	1.453
azopyrimidine					1.349	3.218	1.373	1.914	1.438	1.456

[a] B3LYP/6-311G(2d,p) level calculations.

4. Archive for selected DFT calculations.

1c


```
1\1\GINC-OCTOPUS\FOpt\RB3LYP\6-311G(2d,p)\C12H9F1N3(1+)\PIOTR\29-Apr-2
 013\0\\#P B3LYP/6-311G(2d,p) FOpt(tight) fcheck Geom(nodistance, noang
 le) #P freq(noraman, ReadIso)\\9-F benzotriazinium-4-Me cation, Cs\\1,
 1\C,0.0178059915,0.,0.0073685734\C,0.0040713352,0.,1.4073209964\C,1.20
 4966345,0.,2.0700132519\C,2.413821106,0.,1.3474791116\C,2.3893500442,0
 .,-0.0632379945\C,1.1780623753,0.,-0.7457583291\N,3.5803328104,0.,2.07
 18785321\N,4.7065649147,0.,1.4737055774\C,4.7716483938,0.,0.0999063993
 \C,6.0408565658,0.,-0.4825917856\C,6.2075262131,0.,-1.8529218683\C,5.0
 299434563,0.,-2.6361893706\C,3.7958427135,0.,-2.0605081187\N,3.6485238
 842,0.,-0.6977475948\C,7.5563234026,0.,-2.4989935062\F,-1.1396684945,0
 .,-0.6370054562\H,1.2652785747,0.,3.1504486635\H,-0.9443994001,0.,1.92
 77226719\H,1.0835274257,0.,-1.8210387895\H,6.877220449,0.,0.2035758035
 \H,5.0895126131,0.,-3.7170028497\H,2.8926399188,0.,-2.6467141489\H,7.6
 71482959,0.8784703673,-3.1396793226\H,8.3574864436,0.,-1.7625321239\H,
 7.671482959,-0.8784703673,-3.1396793226\\Version=EM64L-G09RevC.01\Stat
 e=1-A'\HF=-726.7202058\RMSD=5.201e-09\RMSF=5.100e-06\Dipole=0.7574631,
 0.,-1.69817\Quadrupole=15.198246,-15.8407133,0.6424672,0.,-17.2322961,
```

0.\PG=CS [SG(C12H7F1N3),X(H2)]\\

4c-Z

```
1\1\GINC-OCTOPUS\FOpt\RB3LYP\6-311G(2d,p)\C12H9F2N3\PIOTR\29-Apr-2013\
 0\\#P B3LYP/6-311G(2d,p) FOpt(tight) fcheck Geom(nodistance, noangle)
 #P freq(noraman, ReadIso)\\2,4-F2 azopyridine-4-Me cis, C1\\0,1\C,-0.0
 061411562,-0.0000713278,0.0462928849\C,-0.0151048614,-0.1225055246,1.4
 261272362\C,1.1988148844,-0.1346708017,2.0940043743\C,2.4075272993,0.0
 351879764,1.4163129272\C,2.3583792644,0.1328791056,0.025945836\C,1.168
 9827465,0.1126882611,-0.6769437292\N,3.6041758817,-0.1322454407,2.1829
 299587\N,4.6143205451,0.5821480854,2.0855604206\C,4.661321178,1.800472
 6574,1.3346174794\C,5.8777883923,2.0992033972,0.723716429\C,6.01533624
 15,3.3029440582,0.0444275763\C,4.9213729183,4.1717936506,0.0702746209\
 C,3.7687448473,3.7990658064,0.7479965418\N,3.6209323194,2.6274439319,1
 .3674885553\C,7.2828059833,3.6590486179,-0.6839390607\F,3.5065620503,0
 .225551025,-0.6724427806\F,-1.1738726135,-0.010532899,-0.6229832234\H,
 1.2357866506,-0.2646807008,3.1680701448\H,-0.9549047369,-0.2270911053,
 1.9510959522\H,1.1629288106,0.1839643557,-1.7558176618\H,6.6887679206,
 1.3847870909,0.7900381145\H,4.9707092106,5.1378831061,-0.4185254292\H,
 2.921390921,4.4761256277,0.8005297263\H,7.5436021739,4.7085525581,-0.5
 345450706\H,7.1637630756,3.5030734177,-1.7601736345\H,8.1199230532,3.0
 449060706,-0.3507131882\\Version=EM64L-G09RevC.01\State=1-A\HF=-826.79
 60375\RMSD=1.228e-09\RMSF=1.657e-06\Dipole=0.4029946,0.9981221,-0.7190
 948\Quadrupole=2.5565968,0.4933782,-3.049975,5.1994276,-6.6732992,-1.6
 237568\PG=C01 [X(C12H9F2N3)]\\
```

10c-TS

```
1\1\GINC-OCTOPUS\FTS\RB3LYP\6-311G(2d,p)\C12H9F2N3\PIOTR\29-Apr-2013\0
 \\#P B3LYP/6-311G(2d,p) FOpt(QST3) fcheck Geom(nodistance, noangle) #P
  freq(noraman, ReadIso)\\2,4-F2 azopyridine-4-Me cis, C1\\0,1\C,0.0175
 479482,-0.0055450549,0.0162540436\C,-0.0007866297,-0.0284516267,1.4176
 455102\C,1.2119732447,-0.029317196,2.0672326776\C,2.423429207,0.044320
 9145,1.3596510903\C,2.4071275507,-0.0272087887,-0.0738519271\C,1.16153
 45632,-0.02567105,-0.741137217\N,3.566644133,0.255251875,2.0949292732\
 N,4.5712115923,0.9115983451,1.6472178733\C,4.465384116,1.6784411892,0.
 4801350946\C,5.3598382487,2.7548911018,0.371128799\C,5.2410374783,3.66
 55521,-0.6625471508\C,4.1863821884,3.4813489219,-1.575035365\C,3.34174
 27089,2.4042090164,-1.4238983039\N,3.50764372,1.5118217566,-0.44458542
 07\C,6.1850433654,4.8277474223,-0.8057307001\F,3.3680951853,-0.8215458
 571,-0.6476657545\F,-1.1648229317,-0.022847946,-0.6248716594\H,1.27533
 40972,-0.0192051076,3.1485102334\H,-0.945321749,-0.0395039712,1.942240
 795\H,1.113485354,-0.1279382912,-1.8164118453\H,6.1129501832,2.8556499
 702,1.1415869528\H,4.0482659439,4.1598396687,-2.4079196373\H,2.5233211
 648,2.2118317374,-2.1102709246\H,6.6514862699,4.8295552919,-1.79413620
 1\H,6.9745812183,4.7951515811,-0.055445933\H,5.6529508292,5.7767639973
 ,-0.6986203033\\Version=EM64L-G09RevC.01\State=1-A\HF=-826.7629384\RMS
 D=8.270e-09\RMSF=5.049e-06\Dipole=0.2601165,1.7660398,-1.0927513\Quadr
 upole=-0.9755418,1.8275899,-0.8520481,6.8702175,-5.6121195,-2.678175\P
 G=C01 [X(C12H9F2N3)]\\
```

10c

```
1\1\GINC-OCTOPUS\FOpt\RB3LYP\6-311G(2d,p)\C12H9F2N3\PIOTR\29-Apr-2013\
 0\\#P B3LYP/6-311G(2d,p) FOpt(tight) fcheck Geom(nodistance, noangle)
 #P freq(noraman, ReadIso)\\9-F benzotriazinium-4-Me adduct, C1\\0,1\C,
 0.0219240382,0.0036762265,0.016301023\C,0.0062378724,-0.0083300212,1.4
 498524641\C,1.1896293728,-0.0085518562,2.1027821268\C,2.4282382739,0.0
```

202680247,1.3879951218\C,2.4452218842,-0.1921550077,-0.0952999012\C,1.
1258852754,-0.0259877125,-0.7456582654\N,3.5327880722,0.2258378332,2.0
662551288\N,4.6904310554,0.4586211501,1.4463797044\C,4.6660138442,0.73
86029897,0.1444798462\C,5.8359450074,1.2777220183,-0.4575649663\C,5.85
55151065,1.7066712896,-1.7508870016\C,4.6462433684,1.5983340145,-2.502
965983\C,3.5409479242,1.0547723217,-1.9443653852\N,3.5345470181,0.6071
663385,-0.646183202\C,7.0825365836,2.2932222391,-2.3866165587\F,2.8763
967042,-1.5527794146,-0.3306071294\F,-1.1839001729,0.0431696038,-0.582
086308\H,1.2539836204,0.0218658623,3.1827507514\H,-0.947494007,0.00592
17725,1.958856555\H,1.0265952107,-0.0976539711,-1.8183756033\H,6.69843
60581,1.3535144862,0.1909197882\H,4.601800701,1.9361960079,-3.52978532
06\H,2.6169114183,0.947687472,-2.4870387752\H,7.3698906297,1.720943867
8,-3.2732046225\H,7.9254359698,2.3019472489,-1.6963563599\H,6.89703717
09,3.3199462158,-2.7146671274\\Version=EM64L-G09RevC.01\State=1-A\HF=-
826.7891616\RMSD=8.905e-09\RMSF=1.260e-06\Dipole=0.3715638,1.1147974,-
1.6839645\Quadrupole=3.0476271,-4.3169545,1.2693273,3.8610542,-9.59009
73,-3.0229138\PG=C01 [X(C12H9F2N3)]\\

13-Z

1\1\GINC-OCTOPUS\FOpt\RB3LYP\6-311G(2d,p)\C14H12F1N1\PIOTR\23-Jun-2013
\0\\#P B3LYP/6-311G(2d,p) FOpt(tight) fcheck Geom(nodistance, noangle)
#P freq(noraman, ReadIso)\\2-F-phenyl-CHCH-pyridine-4-Me cis\\0,1\C,-
3.5098202455,1.7253921278,0.638245091\C,-3.7913660006,0.3640736688,0.6
778609175\C,-2.9055232292,-0.539950937,0.109454089\C,-1.7058945326,-0.
1272006541,-0.4860752454\C,-1.4747000812,1.2482909699,-0.5245989844\C,
-2.3462300774,2.1714207271,0.0240331849\C,-0.827292743,-1.1276844441,-
1.1125525095\C,0.5049244345,-1.2770059441,-1.0739346691\C,1.5187231755
,-0.5454840488,-0.3056475558\C,2.8695566878,-0.7166935118,-0.635149877
2\C,3.860623207,-0.0570366766,0.082937339\C,3.4418935651,0.7506690601,
1.138563693\C,2.0826571944,0.8544116906,1.4107953682\N,1.1349422105,0.
2310046643,0.7197899629\C,5.3168581426,-0.1938760861,-0.2742752726\F,-
0.3811005093,1.7182234135,-1.1543238471\H,-4.1971157905,2.4412438603,1
.0725627515\H,-3.1287250461,-1.6007127145,0.1357874943\H,-2.1027201648
,3.2238448461,-0.0455912282\H,3.1388068248,-1.3675607013,-1.4599878499
\H,4.1603109832,1.2871021415,1.7479566738\H,1.7406954984,1.470893089,2
.2379268097\H,5.6429153151,0.6462700358,-0.8948362307\H,5.5056304966,-
1.1102505842,-0.8353441093\H,5.9452207696,-0.2016736017,0.6183128806\H
,-1.36763389,-1.8742185851,-1.689563598\H,0.9099216451,-2.0869185207,-
1.6738974554\H,-4.7011258399,0.0078997152,1.1452391773\\Version=EM64L-
G09RevC.01\State=1-A\HF=-695.4860054\RMSD=7.353e-09\RMSF=6.996e-07\Dip
ole=0.5607088,-0.212017,0.0432391\Quadrupole=7.2460054,-1.5827996,-5.6
632058,-0.4110655,-1.4895755,4.4245951\PG=C01 [X(C14H12F1N1)]\\@

13-Z TS

1\1\GINC-OCTOPUS\FTS\RB3LYP\6-311G(2d,p)\C14H12F1N1\PIOTR\24-Jun-2013\
0\\#P B3LYP/6-311G(2d,p) FOpt(QST3) fcheck Geom(nodistance, noangle) #
P freq(noraman, ReadIso)\\2-F-phenyl-CHCH-pyridine-4-Me cis\\0,1\C,-2.
7754206201,1.4607082771,1.0928189764\C,-3.4193723167,0.3351911971,0.54
65818116\C,-2.8368517693,-0.3034452605,-0.5263764964\C,-1.6121235657,0
.1219140249,-1.0680610692\C,-0.9917846599,1.3147113226,-0.5499216649\C
,-1.6052128327,1.953781518,0.5677575795\C,-0.8871578525,-0.6639902786,
-2.0125822161\C,0.4735175604,-0.7001968834,-2.0698410565\C,1.293522667
8,-0.1053106674,-1.0525963977\C,2.6170416299,-0.5333175945,-0.82064215
17\C,3.3534970961,-0.0496586794,0.2413580255\C,2.7272723578,0.86809135
13,1.1068588986\C,1.4324439339,1.2506061613,0.8509672317\N,0.755701127
6,0.8108705589,-0.2128587565\C,4.7675765601,-0.4957752419,0.493607926\

F,-0.5485131499,2.2125831954,-1.5220872716\H,-3.2349469917,1.985571544
,1.9236305366\H,-3.3015004202,-1.1858301207,-0.9553102702\H,-1.1813309
186,2.8849878683,0.9225956177\H,3.0399155107,-1.2767219686,-1.48610892
33\H,3.25615174,1.2863807729,1.9541050126\H,0.8999412442,1.9462418104,
1.4903942758\H,5.4465971996,0.3607760573,0.5149699569\H,5.1137505084,-
1.1854255769,-0.2763257752\H,4.8521395057,-0.9970258998,1.4616011999\H
,-1.4496595322,-1.3472452563,-2.6427520223\H,0.9688672039,-1.363787787
7,-2.7667538959\H,-4.3525622166,-0.0188054441,0.9640269187\\Version=EM
64L-G09RevC.01\State=1-A\HF=-695.4456893\RMSD=4.310e-09\RMSF=1.464e-05
\Dipole=1.3886805,-0.6556405,0.6464334\Quadrupole=4.3771447,-2.7606909
,-1.6164538,0.2111741,0.8234512,6.399751\PG=C01 [X(C14H12F1N1)]\\@

13-Z-cycloadduct

1\1\GINC-OCTOPUS\FTS\RB3LYP\6-311G(2d,p)\C14H12F1N1\PIOTR\24-Jun-2013\
0\\#P B3LYP/6-311G(2d,p) FOpt(QST3) fcheck Geom(nodistance, noangle) #
P freq(noraman, ReadIso)\\2-F-phenyl-CHCH-pyridine-4-Me cis\\0,1\C,-2.
7754206201,1.4607082771,1.0928189764\C,-3.4193723167,0.3351911971,0.54
65818116\C,-2.8368517693,-0.3034452605,-0.5263764964\C,-1.6121235657,0
.1219140249,-1.0680610692\C,-0.9917846599,1.3147113226,-0.5499216649\C
,-1.6052128327,1.953781518,0.5677575795\C,-0.8871578525,-0.6639902786,
-2.0125822161\C,0.4735175604,-0.7001968834,-2.0698410565\C,1.293522667
8,-0.1053106674,-1.0525963977\C,2.6170416299,-0.5333175945,-0.82064215
17\C,3.3534970961,-0.0496586794,0.2413580255\C,2.7272723578,0.86809135
13,1.1068588986\C,1.4324439339,1.2506061613,0.8509672317\N,0.755701127
6,0.8108705589,-0.2128587565\C,4.7675765601,-0.4957752419,0.493607926\
F,-0.5485131499,2.2125831954,-1.5220872716\H,-3.2349469917,1.985571544
,1.9236305366\H,-3.3015004202,-1.1858301207,-0.9553102702\H,-1.1813309
186,2.8849878683,0.9225956177\H,3.0399155107,-1.2767219686,-1.48610892
33\H,3.25615174,1.2863807729,1.9541050126\H,0.8999412442,1.9462418104,
1.4903942758\H,5.4465971996,0.3607760573,0.5149699569\H,5.1137505084,-
1.1854255769,-0.2763257752\H,4.8521395057,-0.9970258998,1.4616011999\H
,-1.4496595322,-1.3472452563,-2.6427520223\H,0.9688672039,-1.363787787
7,-2.7667538959\H,-4.3525622166,-0.0188054441,0.9640269187\\Version=EM
64L-G09RevC.01\State=1-A\HF=-695.4456893\RMSD=4.310e-09\RMSF=1.464e-05
\Dipole=1.3886805,-0.6556405,0.6464334\Quadrupole=4.3771447,-2.7606909
,-1.6164538,0.2111741,0.8234512,6.399751\PG=C01 [X(C14H12F1N1)]\\@

4. References

1. Dunker, M. F. W.; Starkey, E. B. *J. Am. Chem. Soc.* **1939**, *61*, 3005-3007.
2. May, S. W.; Phillips, R. S.; Oldham, C. D. *Biochemistry* **1978**, *17*, 1853-.
3. Carroll, W. A.; Altenbach, R. J.; Bai, H.; Brioni, J. D.; Brune, M. E.; Buckner, S. A.; Cassidy, C.; Chen, Y.; Coghlan, M. J.; Daza, A. V.; Drizin, I.; Fey, T. A.; Fitzgerald, M.; Gopalakrishnan, M.; Gregg, R. J.; Henry, R. F.; Holladay, M. W.; King, L. L.; Kort, M. E.; Kym, P. R.; Milicic, I.; Tang, R.; Turner, S. C.; Whiteaker, K. L.; Yi, L.; Zhang, H.; Sullivan, J. P. *J. Med. Chem.* **2004**, *47*, 3163-3179.
